# Mechanistic Insights into the Cholesterol-dependent Binding of Perfringolysin O-based Probes and Cell Membranes

**DOI:** 10.1038/s41598-017-14002-x

**Published:** 2017-10-23

**Authors:** Benjamin B. Johnson, Mariana Breña, Juan Anguita, Alejandro P. Heuck

**Affiliations:** 1 Program in Molecular and Cellular Biology, University of Massachusetts, Amherst, MA 01003 USA; 2 Department of Biochemistry and Molecular Biology, University of Massachusetts, Amherst, MA 01003 USA; 3 Department of Veterinary and Animal Sciences, University of Massachusetts, Amherst, MA 01003 USA; 4 CIC bioGUNE, 48160 Derio, Bizkaia Spain; 5 Ikerbasque, Basque Foundation for Science, Bilbao, Bizkaia Spain; 6 US Army Natick Soldier Research, Development and Engineering Center, Natick, MA 02115 USA

## Abstract

Cholesterol distribution in the cell is maintained by both vesicular and non-vesicular sterol transport. Non-vesicular transport is mediated by the interaction of membrane-embedded cholesterol and water-soluble proteins. Small changes to the lipid composition of the membrane that do not change the total cholesterol content, can significantly affect how cholesterol interacts with other molecules at the surface of the membrane. The cholesterol-dependent cytolysin Perfringolysin O (PFO) constitutes a powerful tool to detect cholesterol in membranes, and the use of PFO-based probes has flourished in recent years. By using a non-lytic PFO derivative, we showed that the sensitivity of the probes for cholesterol can be tuned by modifications introduced directly in the membrane-interacting loops and/or by modifying residues away from the membrane-interacting domain. Through the use of these biosensors on live RAW 264.7 cells, we found that changes in the overall cholesterol content have a limited effect on the average cholesterol accessibility at the surface of the membrane. We showed that these exquisite biosensors report on changes in cholesterol reactivity at the membrane surface independently of the overall cholesterol content in the membrane.

## Introduction

Cholesterol is essential for the viability of mammalian cells and modulates many membrane properties including fluidity, thickness, and permeability^1^. Cholesterol has been shown to play essential roles in lipid domain formation, segregation of lipids and proteins, and modulation of signal transduction pathways^2^. For these reasons the amount of cholesterol in cellular membranes is tightly controlled^3^, yet its improper regulation has been implicated in several diseases such as atherosclerosis, Niemman-Pick type C disease, and Alzheimer’s disease^2^.

Cholesterol distribution in the cell is maintained by membrane trafficking including the endocytic and exocytic routes, and by non-vesicular transport that presumably use cytosolic lipid transfer proteins^4^. Understanding the interaction of cholesterol with water-soluble molecules is complex given the biphasic nature of the system and the multiple interactions and steric constraints imposed by the composition of the lipid bilayer. The location and orientation of cholesterol in the membrane is determined by the interactions it establishes with other membrane components and membrane-associated molecules^5^. Cholesterol generally locates below the surface of the membrane and orients with its hydroxyl group interacting with hydrophilic phospholipid head groups, and the non-polar rings and acyl tail of cholesterol interacting with the hydrophobic acyl chains of the phospholipids. The interaction of water-soluble molecules with cholesterol is weak on membranes containing low cholesterol given its location inside the membrane^6,7^. However, this interaction increases sharply when cholesterol content increases^8–10^.

Different models have been proposed to explain the mechanism of cholesterol interaction with other lipids and the reactivity of cholesterol in model membranes, such as the condensed-complex model, the super lattice model, or the umbrella model^10^. In addition, it has been proposed that the interaction of cholesterol with water-soluble molecules increases sharply when “active” cholesterol molecules appear in the membrane as a consequence of the saturation of the phospholipid-cholesterol interactions^3^. Other models could also explain this phenomenon^11^. While more work would be required to elucidate which of these models is/are correct, it is clear that there is a sharply increased interaction between water-soluble molecules and cholesterol when the cholesterol content in a membrane bilayer surpasses a certain threshold.

Changes in the phospholipid composition of a membrane at a constant cholesterol concentration (e.g., addition of double bonds or removal of head groups by phospholipases or sphingomyelinases) also increase the cholesterol reactivity with water-soluble molecules^5,12–14^. While these conclusions have been mostly obtained using model membrane systems, the same effects have been shown using cellular systems^7,15,16^. Ultimately, the interaction of cholesterol with water-soluble molecules will be dictated by the cholesterol activity (or effective concentration) in the membrane. The difficulty to determine cholesterol activities (i.e., activity coefficients) in membranes have led researchers to use more qualitative terms to describe the above mentioned effects, for example “cholesterol accessibility” or “active cholesterol”^11,16–19^. In this work, we will use “cholesterol accessibility” to qualitatively describe the effective concentration of cholesterol at the membrane surface. Cholesterol accessibility, or the “ability” of cholesterol to interact with water-soluble molecules at the membrane surface, is modulated by the overall cholesterol content and the composition of the membrane.

Studies of intracellular transport in the cell have benefited from advances in fluorescence microscopy, but detecting lipid transport remains a challenge^20^. Non-polar fluorescent compounds like filipin have been successfully used to determine the localization and transport of total cholesterol in membranes on fixed cells^21^, and fluorescent cholesterol analogs (e.g., dehydroergosterol) have been used to image sterol distribution on live cells^20^. The area where these molecules have been less effective is in detecting changes on the accessibility of cholesterol. Variations in the phospholipid content by hydrolysis of phospholipid head groups or incorporation of double bonds in the acyl chains do not alter the fluorescent signal of filipin or cholesterol analogs (because cholesterol concentration remains unchanged). However, these changes in lipid composition affect cholesterol accessibility^12–14^. The studies of intracellular cholesterol transport require sensors with differential abilities to report not only on total cholesterol content, but also on cholesterol accessibility.

Perfringolysin O (PFO) is a pore-forming protein secreted by the Gram-positive bacteria *Clostridium perfringens* ^22,23^, and is part of a larger family of proteins called cholesterol-dependent cytolysins (CDC)^17^. Our pioneering work with this protein has shown that PFO binding relies on the exposure of cholesterol at the surface of the membrane, and that PFO is a good reporter of cholesterol accessibility on model membranes and mammalian cells^12,13,24,25^. Single-Cys derivatives of PFO can be specifically labeled with fluorophores (or other probes) and employed to report on cholesterol accessibility on the plasma membrane^25^. Thus, it is not surprising that the interest in the use of PFO and other CDC to study cholesterol in membranes has grown in recent years^11,16,26–31^. However, multiple factors can affect the interaction of PFO with membranes, and detailed studies on this protein-membrane interactions are needed to properly interpret and compare the results obtained with these emerging palette of cholesterol biosensors.

In this work, we obtained and employed a non-lytic PFO derivative to selectively study cholesterol accessibility on live cells. Two substitutions in domain 3 (D3) were necessary and sufficient to completely abolish the lytic activity of the probe on cells. The binding affinity of these non-lytic PFO for cholesterol was modulated by modifications in D3. We also identified single amino acid substitutions in domain 4 (D4) loops which increased or decreased the affinity of this non-lytic PFO derivative for cholesterol at the surface of the membrane. Using these PFO-based probes we found that cholesterol accessibility at the plasma membrane of live RAW264.7 cells was maintained fairly constant when incubated with methyl-β-cyclodextrin (mβCD)/cholesterol mixtures, a treatment that is commonly used to modify the cholesterol content of cell membranes. These biosensors are essential to study the role of cholesterol accessibility in cholesterol homeostasis independently of the total cholesterol content of the samples.

## Results

### Engineering a non-lytic PFO probe to measure cholesterol accessibility on live cells

We have previously shown that cholesterol exposure at the membrane surface is required and sufficient to trigger PFO binding and all the structural re-arrangements that lead to oligomerization^12,13,24^. The specificity of PFO for cholesterol^24,32^, and the ability to modify the cholesterol affinity of PFO using single amino acid modifications^13^ prompted us to develop PFO-based probes to study cholesterol in cell membranes^25^. Non-lytic probes are essential to preserve membrane integrity when studying cholesterol localization and transport in live cells. Different approaches have been used to inactivate PFO, including proteolytic cleavage, site-directed mutagenesis, and isolation of just the membrane-binding domain (D4)^25,33^. However, little is known on how these modifications affect cholesterol recognition and binding.

**Figure 1:**
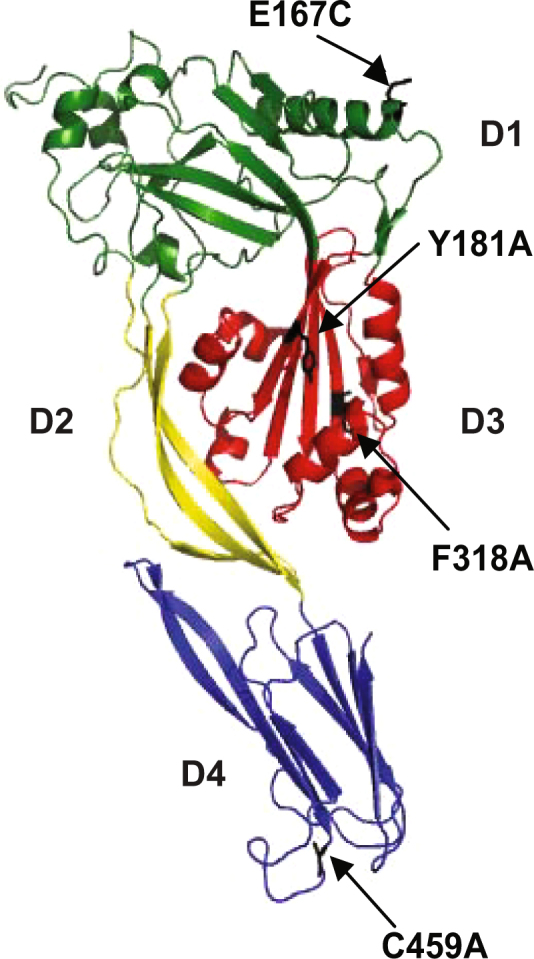
Molecular structure of monomeric water-soluble PFO. Crystal structure of PFO in a ribbon representation showing the four amino acid substitutions contained in the pPFO parental derivative. The E167C and C459A substitutions allow for a Cys specific labeling of the PFO derivative. The Y181A and F318A substitutions produce a non-lytic PFO derivative. Domains (D) are color coded and numbers indicated (PDB ID: 1PFO).

The _F_PFO derivative used in our original studies^25^ contained the following three substitutions: C459A eliminates the unique Cys in native PFO, E167C introduces a unique Cys on a non-membrane interacting domain for specific labeling, and F318A eliminates pore forming activity on liposomes (Table 1, Fig. 1 ^34^). Additional amino acid substitutions to PFO derivatives defined in Table 1 are indicated in this work as superscripts. Two derivatives, _F_PFO^D434S^ and _F_PFO^L491S^, showed 10 mol% difference in the cholesterol threshold. Cholesterol threshold is defined as the cholesterol concentration at which the increase in Trp emission of PFO is half of the emission when binding is complete^25^.

**Table 1:** Nomenclature for abbreviations used for PFO derivatives.

Name	Contained Modifications
nPFO	no modifications^a^
rPFO	C459A
_F_PFO	E167C, F318A, C459A
pPFO	E167C, Y181A, F318A, C459A

^a^Recombinant His-tagged-PFO with wild type sequence expressed using pAH11 plasmid^12^.

When employing these PFO derivatives to study cholesterol accessibility on live cells (rather than on fixed cells), we noticed considerable loss of cell membrane integrity in the samples. This integrity loss was intriguing due to the inability of _F_PFO to form pores in liposomes^34^. This unexpected observation prompted us to perform a thorough characterization of these PFO derivatives that are often assumed to be non-lytic on cells (e.g.^31^).

**Figure 2:**
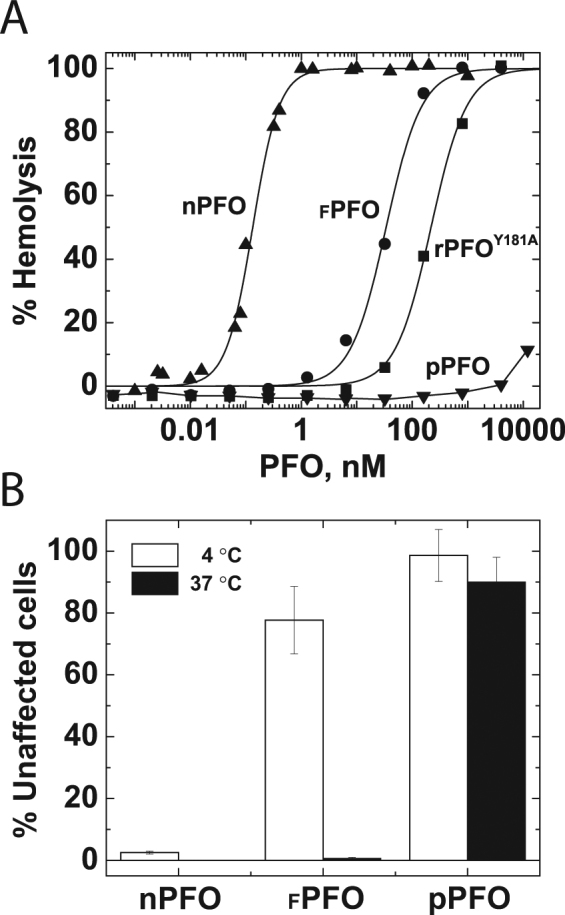
Two substitutions in D3 are required to abolish PFO cytolysis. (**A**) hemolytic activity of PFO. The indicated amount of the nPFO (filled triangles), _F_PFO (filled circles), rPFO^Y181A^ (filled squares), and pPFO (filled inverted triangles) was incubated with a 0.5% suspension of stacked RBC for 20 min at 37 °C in a 96 well plate (final volume 200 µl). Percent hemolysis was determined by measuring hemoglobin release post-incubation using absorbance at 540 nm in a plate reader. Values were corrected by spontaneous release of hemoglobin using a sample without PFO. Total hemoglobin released was determined by osmotic lysis of RBC with water. (**B**) quantification of cells with intact membranes after incubations with the indicated PFO derivatives for 20 min at 37 °C (filled bars) or 4 °C (open bars). The graph shows the percentage of cells that did not incorporate trypan blue after incubation with the indicated PFO derivative (final concentration 1 µM) when compared to cells that were not exposed to the PFO derivative. Values correspond to the average of 3 determinations and standard deviation.

Hemolysis of red blood cells (RBC) constitutes a good measure to evaluate how amino acid substitutions affect the cholesterol-dependent cytolytic activity of PFO derivatives on natural membranes. To determine the effect of amino acid substitutions on the activity of PFO, we compared the hemolytic activity of derivatives containing single aromatic substitutions in D3 (_F_PFO, rPFO^Y181A^), and a derivative containing both F318A and Y181A substitutions (named pPFO, see Table 1). Not surprisingly the PFO derivatives with a single aromatic substitution in D3, _F_PFO and rPFO^Y181A^, were less lytic than nPFO. The concentration yielding 50% hemolysis (HA_50_) for nPFO, _F_PFO, and rPFO^Y181A^ were approximately 0.2 nM, 30 nM, and 250 nM, respectively (Fig. 2A). Only pPFO showed minimal hemolytic activity, with an estimated HA_50_ much higher than 10 μM (six orders of magnitude higher than that of nPFO).

The effect of PFO derivatives on cell membranes was also evaluated on nucleated RAW 264.7 cells. The integrity of the plasma membrane in this macrophage-like cell line after incubation with nPFO, _F_PFO, or pPFO was determined by the incorporation of trypan blue. Both _F_PFO and nPFO caused considerable loss of cell integrity at 37 °C, in contrast to pPFO that did not affect the membrane integrity at any temperature in the 4–37 °C range. The activity of _F_PFO and nPFO differed when the incubation was done at low temperature (4 °C, Fig. 2B), where only nPFO affected the membrane. nPFO is not expected to form pores at 4 °C^24,35^, however cell integrity was compromised when nucleated cells were incubated with nPFO at this temperature. This effect was likely caused by the insertion of membrane bound pre-pore complexes during the brief exposure of cells to room temperature (~20–23 °C), at the time of inspection in the microscope. Based on these observations, it is clear that pPFO did not affect the integrity of cell membranes and therefore constituted a good framework to generate sensors with different cholesterol thresholds for the study of cholesterol accessibility on live cells.

### Amino acid substitutions in domain 3 modify the cholesterol-dependent binding of PFO derivatives

**Figure 3:**
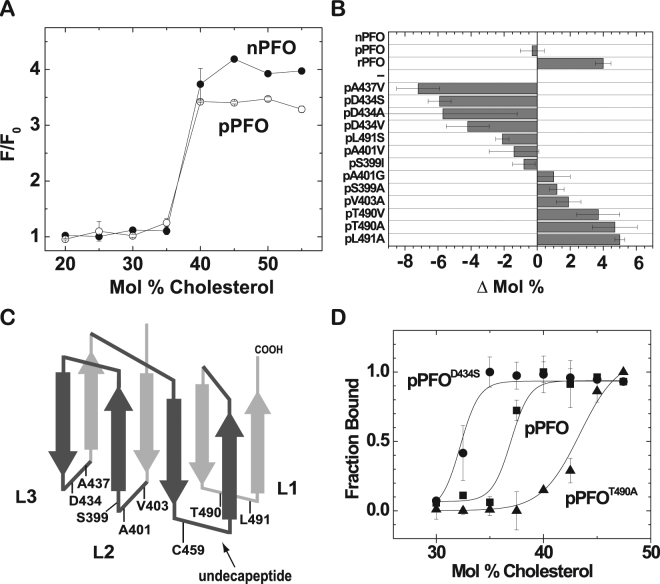
Generation of pPFO derivatives with different thresholds for cholesterol binding. (**A**) cholesterol-dependent binding isotherms for nPFO and pPFO. PFO derivatives (final concentration of 0.2 μM) were incubated with liposomes of varying cholesterol concentrations and a constant 1:1:1 molar ratio of POPC, POPE, and SM (final total lipid concentration of 0.2 mM). PFO binding was determined using the intrinsic Trp emission before (F_0_) and after addition of liposomes (F). (**B**) relative change on the cholesterol threshold for different pPFO derivatives was calculated using the threshold for nPFO obtained with the exact same liposomal preparation. The difference between the cholesterol threshold of a pPFO derivative and nPFO was represented by Δmol % as defined in the text. Positive value indicates the need for more cholesterol to trigger binding, and negative value indicates the need for less cholesterol. The letter p preceding the amino acid substitution indicates the use of pPFO. Unless otherwise indicated, the cholesterol thresholds were calculated by fitting the binding isotherms using a Boltzmann sigmoid function and the OriginPro 8.6 software. Error bars indicate standard errors calculated by the fitting procedure. Values for pPFO^D434S^ and pPFO^T490A^ correspond to the average and the standard deviation of three and two independent cholesterol threshold determinations, respectively. (**C**) a cartoon depiction of the two β-sheets that constitute D4 (dark and light grey). The loops (L) that interact with the membrane, the conserved undecapeptide, the C-terminus, and the location of amino acids substituted in this work are indicated. (**D**) cholesterol-dependent binding isotherms for the PFO derivatives selected for further live cell testing, pPFO^D434S^ (filled circles), pPFO (filled squares), and pPFO^T490A^ (filled triangles). Binding measurements were done as indicated in A. Lines correspond to the fitting done as indicated in B. Values at each cholesterol mol % are the average of 3 measurements and the errors bars indicate the standard deviation.

As described above, modifications to the PFO D4 alter the cholesterol threshold of the protein^25^. When the F318A change was introduced in the distal D3 of the protein to generate the _F_PFO derivative (Fig. 1), no variations in the cholesterol threshold were observed^25^. The substitution of a second aromatic residue (Y181A) in D3 unexpectedly reduced the cholesterol threshold of the protein to levels like those observed for the wild type nPFO (Fig. 3A). Given the long distance that separates Y181 from the D4 tip, it is clear that modifications outside the conserved loops can alter the cholesterol binding properties of PFO derivatives, presumably through the coupling between D3 and D4^36,37^. We have therefore generated a “parental” non-lytic PFO derivative (named pPFO, Table 1), with the same cholesterol threshold as the wild type protein (Fig. 3A).

### Tuning the pPFO cholesterol threshold by amino acid substitutions in the membrane binding domain

To quantify the effect that amino acid substitutions have on the cholesterol threshold of PFO, we defined the term “Δmol% cholesterol at half binding” or just “Δmol%” for simplicity. This is the difference in mol% cholesterol between the concentration of cholesterol required for half binding for the PFO derivative under analysis, and the one for nPFO (used as a reference) measured using the same membranes. The use of a relative quantity (Δmol%), instead of the actual mol% at half binding, eliminated the differences that may arise from small variations in the lipid composition of different preparations of liposomes. In our hands, the absolute cholesterol mol% at half binding for a PFO derivative could vary approximately up to 3 mol% when two independent liposome preparations are compared^25^. These variations are caused by small differences in sample handling when preparing liposomes and lipid solutions. It is therefore important to emphasize that to determine a Δmol%, the binding isotherms (samples and reference) need to be obtained using the same liposome preparation. A PFO derivative that requires more cholesterol for binding than nPFO will show a positive Δmol% value, while a negative Δmol% will indicate a derivative that requires less cholesterol for binding. For example, the Δmol% for rPFO is +4, indicating that for half binding this derivate will required 4 mol% more cholesterol in the membrane than nPFO (Fig. 3B).

Our previous studies using _F_PFO showed that the Δmol% for _F_PFO^D434S^ and _F_PFO^L491S^ were approximately −5, and +5, respectively^25^. We analyzed the effect of introducing the same D434S or L491S substitutions in pPFO. As expected, pPFO^D434S^ showed a Δmol% of −5.5 (Fig. 3B). In contrast to the positive Δmol% observed for _F_PFO^L491S^,^25^ pPFO^L491S^ showed a negative Δmol% of −1.5, not significantly different than the one for the parental pPFO. The single Y181A change in _F_PFO^L491S^ was sufficient to reverse the effect caused by the L491S modification on _F_PFO binding. This result corroborated that the cholesterol binding properties of PFO are not only affected by substitutions of amino acids located at the loops of D4, but also by substitutions of amino acids located in the distal D3.

We initiated a scan for amino acid substitutions that reduced or increased the Δmol% of the non-lytic pPFO. Several amino acids located in D4 loops were modified (Fig. 3C), and the effect of the side chain substitutions on the Δmol% determined. The contribution of certain amino acid side chains to the cholesterol threshold of pPFO was evaluated by modifying a native amino acid to Ala or Gly. Elimination of the negative charge on D434 in loop 3 (L3) produced a large and negative Δmol% independently of the size or polarity of the side chain. A similar effect was observed if a polar Ser, a nonpolar and bulky Val, or a nonpolar and small group Ala substitution was introduced in place of D434 (Fig. 3B). Introduction of a non-polar side chain in L3 (A437V) also produced a decrease in the cholesterol threshold (Δmol% = −3.5), suggesting that substitutions that favor the interaction/insertion of this loop with/into the hydrophobic core of the membrane may play an important role on the cholesterol-dependent binding of PFO.

When the contribution of non-polar groups was tested in L2, a similar but smaller effect was observed when V403 was replaced by Ala (Δmol% = 2). Substitutions of other residues in this loop showed no significant effects on the cholesterol threshold of the protein, indicating that the interaction/insertion of L2 is less critical for the cholesterol-dependent binding of PFO. Neither eliminating the methyl group (A401G) nor introducing a larger non-polar group (A401V) had significant effects on the cholesterol threshold of pPFO (Fig. 3B). Similarly, substitutions of S399 to Ala or Ile had no effect on pPFO cholesterol threshold (Fig. 3B).

In contrast, major effects were observed when either T490 or L491 on L1 were replaced with Ala (Δmol% = +4.5, Fig. 3B). These results were not surprising since these two amino acids were proposed to be the cholesterol recognition motif for PFO^38^. Substitution of the hydrophobic L491 by Ser, or the substitution of the hydrogen bond-former T490 for the non-polar and bulky Val showed only minor effects on the Δmol% of pPFO. These results suggested that while important for cholesterol interaction, these two residues alone cannot constitute the sole binding motif for cholesterol.

Based on the above results, two pPFO derivatives were selected for the studies of cholesterol accessibility on live cells: pPFO^D434S^ and pPFO^T490A^. These derivatives showed lower and higher cholesterol thresholds, respectively, when compared with the parental pPFO (Fig. 3D).

### PFO derivatives showed a similar saturation binding isotherms on cell membranes or model membranes

**Figure 4:**
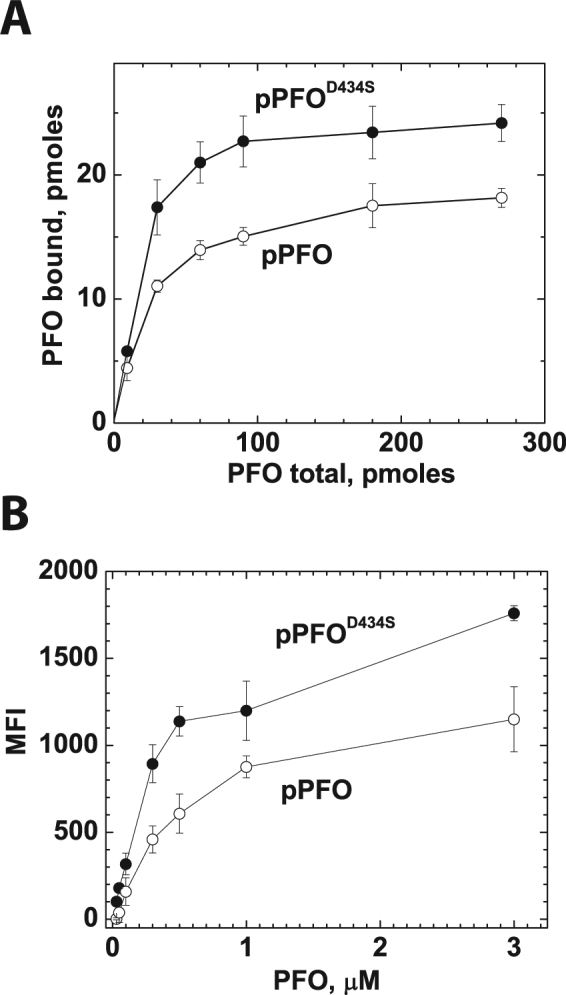
Binding saturation isotherms for pPFO and pPFO^D434S^ when incubated with liposomes or RAW264.7 cells. (**A**) concentration dependent binding of pPFO (open circles) and pPFO^D434S^ (filled circles) to liposomes containing 38 mol% cholesterol and POPC, POPE, and SM in a constant 1:1:1 molar ratio (final total lipid concentration 10 μM). Samples containing increasing amounts of pPFO or pPFO^D434S^ were incubated with liposomes for 20 min at 37 °C in a final volume of 300 μL. Binding was determined as indicated in methods. (**B**) concentration-dependent binding of Alexa488-labeled pPFO and pPFO^D434S^ (20 min incubation at 4 °C) to live RAW264.7 cells determined using flow cytometry as described in methods. Data points are the average of at least two measurements and their standard deviation.

Model membranes are very useful to study protein-membrane interactions, particularly to discriminate the contribution of individual membrane lipids. Plasma membranes differ from model membranes in many aspects, for example the asymmetry of lipids in each leaflet, alterations in membrane curvature, and the presence of a membrane potential, proteins, lipid domains, sugars, etc. To compare the cholesterol-dependent binding of the PFO derivatives between liposomes and cell membranes, we determined the binding isotherms on RAW 264.7 cells and on liposomes made with 38 mol% cholesterol. The binding saturation isotherms for pPFO^D434S^ and pPFO were similar when using cell or liposomal membranes (Fig. 4). The binding of pPFO^D434S^ was always higher than pPFO, independent of the concentration of the protein. At higher protein concentrations a stable saturation level for binding was difficult to obtain with live cells because the internalization of the probe via endocytosis may interfere with the fluorescent signal determinations, even when low temperatures were used to inhibit this process (Fig. 4B). It is clear from these data that the introduction of the D434S substitution increased the number of pPFO molecules that can bind to a membrane at a particular cholesterol concentration. Moreover, the differential binding among PFO derivatives was not affected by other factors present in natural membranes (e.g., glycolipids, membrane potential, proteins, etc.).

### PFO binding decreased the cholesterol accessibility at the membrane

One explanation for the differential binding observed for pPFO and pPFO^D434S^ in model and natural membranes (Fig. 4) is that the pPFO derivative binds more cholesterol molecules than pPFO^D434S^. If this is the case, for the same number of cholesterol molecules one would expect more pPFO^D434S^ molecules associated with the membrane than that of pPFO. Alternatively, if both proteins bind the same number of cholesterol molecules, the differential binding could be explained by the different cholesterol threshold. Cholesterol accessibility at the membrane is progressively reduced by the binding (and sequestration of cholesterol) of each protein molecule. Binding of a PFO derivative will continue up to the point where the accessibility of cholesterol drops below its particular binding threshold, for example, below 35 mol% cholesterol for the model membrane-PFO combination used in Fig. 3A.

A sequential binding assay was used in order to distinguish whether each PFO derivative binds a different number of cholesterol molecules or if PFO derivatives decreased cholesterol accessibility upon binding. In this assay, the membrane is first saturated with a PFO derivate that requires more cholesterol (PFO_1_), and the binding of a second derivative that requires less cholesterol (PFO_2_) is subsequently evaluated on the membrane saturated with PFO_1_. If the PFO derivatives bind different number of cholesterol molecules, no binding will be observed for PFO_2_ because all accessible cholesterol molecules will be bound to PFO_1_. In contrast, if PFO_1_ binding decreased the overall cholesterol accessibility, at saturation, the cholesterol accessibility at the membrane will be just below the binding threshold for this derivative. Derivatives that require less cholesterol accessibility though will still be able to bind.

**Figure 5:**
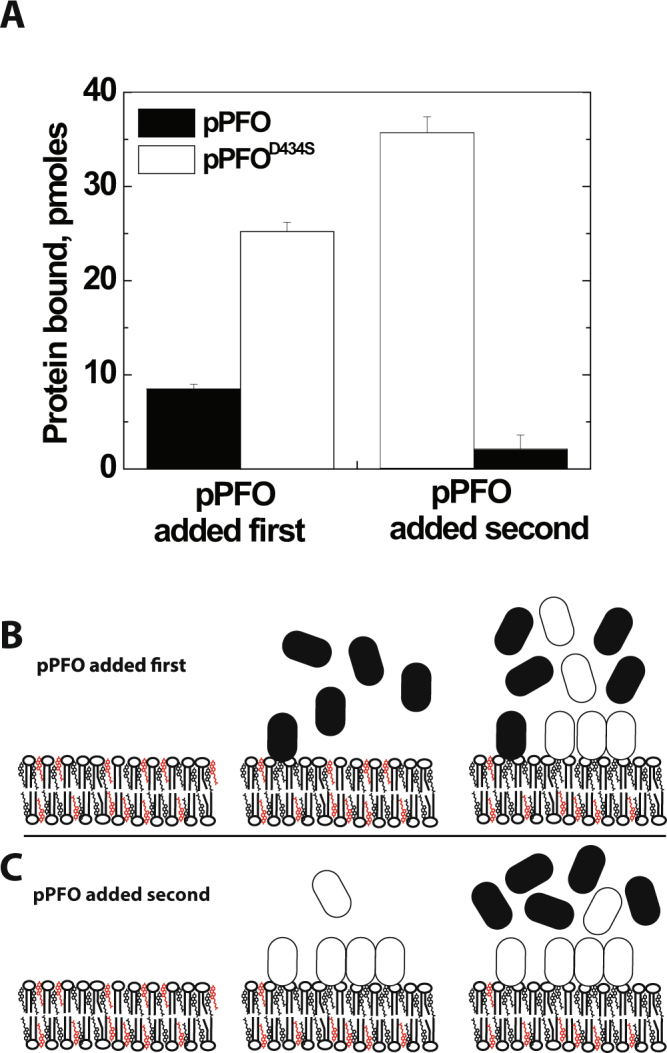
Sequential binding of pPFO derivatives showed that PFO binding reduced cholesterol accessibility. (**A**) pPFO added first (left). Liposomes with cholesterol content just above the cholesterol threshold for pPFO were saturated with pPFO (100 nM protein, 100 µM total lipids) and incubated for 20 min at 37 °C. After equilibration, bound protein was quantified using intrinsic Trp fluorescence as indicated in methods (black bar). Subsequently an equimolar amount of pPFO^D434S^ was added to the sample, incubated for additional 20 min at 37 °C, and bound protein determined (white bar). pPFO added second (right). The experiment was done as indicated above, but the order of addition of the PFO derivatives was inverted. Bars represent the average of three measurements and error bars the standard deviation. (**B**) from left to right, cartoon representation of the membranes before, after addition of pPFO (black ovals), and after addition of pPFO^D434S^ (white ovals). (**C**) from left to right, cartoon representation of the membranes before, after addition of pPFO^D434S^ (white ovals), and after addition of pPFO (black ovals). Cholesterol accessibility is schematically represented by the number of red cholesterol molecules, the higher is the number the highest is the accessibility.

The sequential binding assay was done using pPFO and pPFO^D434S^, two derivatives that showed a ~5 mol% difference in their Δmol% on model membranes (Fig. 3D). To maximize visualization of any variation in pPFO binding, liposomes containing ~38 mol% cholesterol were used. Based on the isotherm showed in Fig. 3D, only 30–40% of the added pPFO derivative will bind to these membranes, while saturation levels are expected for the pPFO^D434S^ derivative. Binding was determined using the change in Trp emission intensity that follows membrane interaction (Fig. 5). Saturation of the membrane with pPFO did not eliminate the binding of the pPFO^D434S^ derivative (Fig. 5A and B). In contrast, when the addition of the derivatives was done in the reverse order, saturation with the pPFO^D434S^ blocked the binding of pPFO (Fig. 5A and C). These results suggested that binding of pPFO reduced cholesterol accessibility to the point where this derivative can no longer bind. Since the pPFO^D434S^ has a lower cholesterol threshold, it could bind to membranes saturated with pPFO. Moreover, the total amount of toxin bound (pPFO and pPFO^D434S^) in each experiment was similar independently of the order of addition. The amount of pPFO bound when it was added first was equivalent to the reduction in binding observed for pPFO^D434S^ (compare open bars in Fig. 5). These data support a model where the subsequent binding of PFO molecules reduces the cholesterol accessibility at the membrane surface.

### Incubations of cells with methyl-β-cyclodextrin:cholesterol complexes resulted in only moderate changes in cholesterol accessibility

Two non-lytic PFO derivatives were labeled with Alexa488 and they were tested for their ability to bind to nucleated cells using flow cytometry. Modification with Alexa488 did not alter the cholesterol threshold of the proteins (Fig. S1). In order to vary the cholesterol content in natural membranes, RAW 264.7 cells were incubated with mβCD:cholesterol complexes^39,40^. For all incubations the concentration of mβCD was maintained constant at 5 mM to account for any non-specific effect of the reagent (e.g., interactions with other lipids). Different cells when incubated with 0.5–5 mM mβCD have shown a depletion of 30–40% of un-esterified cholesterol^40^. Similarly, incubations with mβCD:cholesterol have shown to enrich cholesterol levels up to 50%^40^. Many factors can affect the depletion/enrichment when using these treatments, for example type of cell, cell density, temperature, etc. It is well documented that cells will have varied amounts of unesterified cholesterol at the plasma membrane, depending on the mβCD:cholesterol ratio used. Our goal in these experiments was to vary the cholesterol levels on live cells, and to evaluate how the PFO derivatives respond to these variations. The overall content of cholesterol in the plasma membrane was not determined because quantification of the overall cholesterol content will only corroborate the well established effect that cyclodextrin:cholesterol incubations have on membranes (e.g., increase or decrease to the total cholesterol content), but not changes on cholesterol accessibility. The PFO-based probes were not designed to quantify cholesterol content but to report on changes on cholesterol accessibility on live cells.

**Figure 6:**
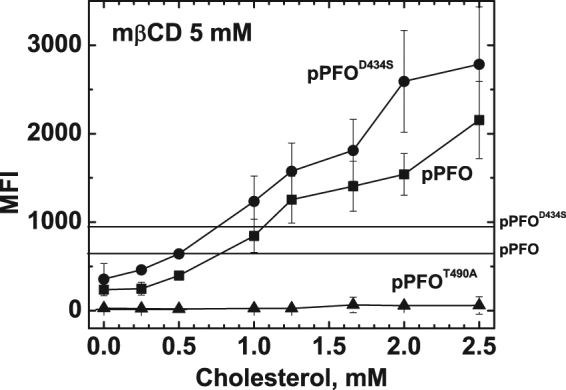
Differential binding of pPFO derivatives to RAW264.7 cells with altered cholesterol content. Quantification of binding of Alexa488-labeled pPFO^D434S^ (filled circles), pPFO (filled squares), and pPFO^T490A^ (filled tringles) to RAW264.7 cells treated with varied amounts of cholesterol complexed with 5 mM mβCD (1 h incubation at 37 °C). Treated cells were incubated with each PFO derivatives (0.5 µM protein, for 20 min at 4 °C) and binding was quantified using flow cytometry as described in methods. Binding of pPFO^D434S-Alexa488^, pPFO^Alexa488^, and pPFO^T490A-Alexa488^ to untreated cells was 873 ± 61, 629 ± 86, and 44 ± 6, respectively, and is indicated by a horizontal line. The pPFO^T490A-Alexa488^ derivative showed no significant binding. Independently of the cholesterol content, binding of pPFO^D434S-Alexa488^ to live cells was always higher than that for pPFO^Alexa488^. Data points are the average of three measurements and their standard deviation.

The binding of pPFO^D434S-Alexa488^ to untreated cells (determined as the mean fluorescence intensity, MFI), was 30% higher than that observed for pPFO^Alexa488^ (Fig. 6, horizontal lines). No significant binding was observed for pPFO^T490A-Alexa488^. When the binding of these three derivatives was determined in cells treated with different cholesterol:mβCD ratios, a similar response was observed. Binding of pPFO^D434S-Alexa488^ was higher than the one for pPFO^Alexa488^, and no significant binding for the pPFO^T490A-Alexa488^ was detected. For pPFO^D434S-Alexa488^ and pPFO^Alexa488^, the binding to cells treated with the highest cholesterol concentration was 3.3 fold higher than the one observed on non-treated cells. When cells were treated with 5 mM mβCD alone (lower cholesterol content), the binding was 60% lower than the one observed on untreated cells.

When these results are compared with the ones obtained with model membranes (Fig. 3D), the average cholesterol accessibility on live cells with different cholesterol levels was similar to that observed for liposomes prepared with 35–40% cholesterol. Both pPFO^D434S-Alexa488^ and pPFO^Alexa488^ were able to bind, but not pPFO^T490A-Alexa488^. The overall binding, for both pPFO^D434S-Alexa488^ and pPFO^Alexa488^ derivatives, increased when cells were treated with solutions containing more than 1 mM cholesterol, and decreased with solutions containing less than 1 mM cholesterol (Fig. 6). The apparent linear response was caused by the saturation amounts of PFO derivatives used in this assay. Similar effects were observed using model membranes. The typical sigmoidal shape of the isotherm (Fig. 3A) become linear at high protein concentrations (Fig. S2). Taken together, these results suggest that cholesterol accessibility at the plasma membrane of live RAW264.7 cells was maintained fairly constant after treatments with cholesterol:mβCD to increase and decrease the overall cholesterol content.

## Discussion

The binding properties of the cholesterol-dependent cytolysins have been utilized in recent years to visualize and study cholesterol in membranes (reviewed in^41–43^). While it was initially claimed that PFO binds to cholesterol in “membrane rafts”^41^, it has become apparent that exposure of cholesterol at the membrane surface is the key factor that triggers toxin-membrane interaction^11–13,16,24^.

The characterization of PFO-cholesterol interaction have been done mostly using full-length protein (review in^17^), but in recent years the use of isolated D4 as a cholesterol probe has increased^28,30,44^. While D4 is small and non-lytic^45^, the limited characterization done on isolated D4 suggests that its cholesterol-dependent binding properties differ from the ones observed for the full-length protein^44^. The full length PFO binding is highly cooperative, going from no-binding to full-binding in a narrow range of cholesterol concentrations (~10–15 mol %)^11,12,16,46^. In contrast, the binding of most D4-based probes increases more gradually with the increase in cholesterol concentration (supplementary Figures in^28,30^). While it is clear that the binding properties of full-length versus D4-based probes differ, it has recently been shown that localization of full length probes are not affected by oligomerization when compared with D4-based probes^31^. Both full-length and D4-based probes could provide important and complementary information about cholesterol content and accessibility at the membrane surface. The effective use of PFO as a cholesterol-sensing probe requires a precise understanding of the cholesterol-toxin interactions.

In this work we unveiled some fundamental aspects of the cholesterol-dependent binding of the toxin and obtained a non-lytic PFO-based biosensor to analyze cholesterol accessibility on live cells. First, the lytic properties of PFO derivatives on liposomes differ from the ones observed on live cells (Fig. 2). Second, amino acid substitutions in D3 (far away from the membrane interacting domain) can alter the cholesterol-dependent binding of PFO derivatives (Fig. 3A). Third, modifications that favor the interaction of L3 with the membrane (i.e., charge elimination or increase in hydrophobicity) reduced the cholesterol threshold of PFO derivatives. In contrast, modifications to L1 or L2 had little effect or increased the threshold (Fig. 3). Fourth, the saturation binding observed for PFO on model membranes was very similar to the one observed on live cells, suggesting that binding is only modulated by cholesterol accessibility and not affected by components present in cell membranes and absent in liposomes (Fig. 4). Fifth, binding of PFO molecules reduced cholesterol accessibility at the membrane surface (Fig. 5). Finally, we found that the treatment of live RAW 264.7 cells with mixtures of mβCD:cholesterol had limited effect on the average cholesterol accessibility at the membrane surface (Fig. 6).

These findings are important because a precise characterization of PFO-based biosensors is essential to interpret the data obtained using flow cytometry, fluorescence microscopy, or other techniques. For example, PFO derivatives with a single aromatic modification in D3 have been assumed to be non-lytic (e.g., Y181A^31^), but based on the results presented in this work it is clear that cell lysis and concomitant internalization of the probes may occur when using derivatives with single aromatic modifications. Another potential problem when using probes prepared by proteolytic inactivation of PFO and biotinylation^47^ or by iodination of PFO^27^ is that these modifications may alter the cholesterol-dependent binding of the protein, as shown in this work for changes in D3 (Figs 2 and 3). The extent of biotinylation of amine groups and iodination are difficult to control and quantify, therefore the cholesterol binding properties of each protein preparation should be carefully characterized before use. For example, they should be compared to the wild type protein using the same membranes as suggested in this work.

Previous studies have reported that the substitution of Y181A or F318A introduced into rPFO interferes with the proper alignment of the β-hairpins in D3 that extend to form the β-barrel^34,48^. Both _F_PFO and rPFO^Y181A^ form circular pre-pore complexes, but they are not able to insert the transmembrane-hairpins to perforate liposome membranes^34,48^. Characterization of PFO derivatives using RBC showed lower hemolytic activity for rPFO^Y181A^ ^48^ and _F_PFO when compared to the activity for rPFO or nPFO (Fig. 2A). At concentrations commonly used to study the interaction of PFO with cell membranes (high nM to low μM range^25,27,28,38,49^), both rPFO^Y181A^ and _F_PFO showed membrane disruption and a double aromatic to Ala substitution was required to eliminate this phenomenon.

Novel aspects about the PFO pore-formation mechanism were unveiled during the characterization of the thermal-dependent lysis of different PFO derivatives (Fig. 2). In addition to assisting with the alignment of the transmembrane hairpins between adjacent PFO monomers^34^, the Y181 and Y318 aromatic residues also play a role in the energetics for the unfurling of the short alpha helices to extended β-hairpins in D3. Temperature plays a key role for the pre-pore to membrane-inserted complex transition^35^. It is well established that at low temperatures (2–4 °C), PFO oligomerization occurs, pre-pores are formed, but no pore formation is observed when the toxin is incubated with model or cell membranes^26,35,50^. A similar effect is observed when aromatic residues located in D3 are substituted to Ala, presumably by interfering with the alignment of the β-strands required for insertion of the β-hairpins^34^. Here, we showed that aromatic substitutions in D3 changed the temperature-dependent membrane disruption of PFO on RAW 264.7 cells (Fig. 2B). In contrast to nPFO, _F_PFO only caused disruption when samples were incubated at 37 °C. These results suggest that the F318A substitution increased the activation energy for the pre-pore to pore transition (i.e., insertion of the β-hairpins), but did not abolish membrane insertion. This observation is in good agreement with the results obtained using a single aromatic substitution in D3, Y181A^27^. Removal of both aromatic residues was required to abolish pore formation at 37 °C (Fig. 2A), the temperature where physiological processes take place in humans and other mammals.

While resulting in the non-lytic pPFO derivative, the introduction of the Y181A mutation into _F_PFO had some unforeseen results. In contrast with the neutral effect that the F318A substitution had on the cholesterol binding threshold of rPFO^25^, the addition of the Y181A substitution to _F_PFO decreased the cholesterol threshold of _F_PFO to the one observed for wild type nPFO (Fig. 3A). It is worth noting that the Y181A substitution by itself into rPFO is not enough to alter the cholesterol threshold^27^. While the molecular mechanism by which a double aromatic substitution in the distant D3 altered PFO binding to membranes remains uncertain, these results are in good agreement with the proposed interplay between D3 and D4 on PFO cholesterol-dependent binding^24,36^.

We introduced the use of Δmol% (as defined above) to characterize the cholesterol-dependent binding among PFO derivatives. The cholesterol concentration at which 50% of binding is achieved is a good reporter for the binding properties of different PFO derivatives^25^. Absolute determinations of this value require a precise quantification of the cholesterol concentration present in each batch of membranes^25^. The use of a relative measurement for protein affinity, instead of an absolute measurement, simplifies the characterization of PFO derivatives and facilitates the comparison of results obtained using different membranes and/or procedures to prepare liposomes (e.g., see refs^12,44,46^). Variations on the lipid composition of the membrane (e.g., saturation of the acyl chains or phospholipid head groups) affect the cholesterol-dependent binding of PFO^12^, nevertheless the relative binding affinities among PFO derivatives are not affected (see refs^13,25^).

We evaluated a series of individual amino acid changes in L1, L2, and L3, with the goal of obtaining pPFO derivatives with increased and decreased cholesterol thresholds with respect to the threshold observed for pPFO (Fig. 3). In general, modifications on the well conserved L1 increased the cholesterol threshold, with the exception of L491S. Replacing a long hydrophobic Leu for a shorter hydrogen bond former Ser slightly decreased the amount of cholesterol required to trigger binding. A single amino acid substitution (L491S or T490V) to the proposed cholesterol binding motif of PFO T490-L491^38^, showed little effect on the cholesterol-binding properties of pPFO, a derivative with similar binding properties than the wild type toxin.

Modifications to L3 showed negative Δmol% values (Fig. 3). In particular substitutions in D434^25^, as recently confirmed by Farrand *et al*.^49^. The mechanism by which these amino acid substitutions alter PFO binding is not completely understood, but it has been suggested that at least for substitutions on the D434, the solvation energy of the side chain of the amino acid may be responsible of the changes in binding affinities observed for different PFO derivatives^49^. When the ΔΔG of the water-octanol partition^51^ for side chain modifications introduced in D4 are compared with the obtained Δmol% values (Table 2), a correlation was observed for substitutions in L2 (A401, V403) and L3 (D434, A437). No correlation was found for substitutions on C459, T490, or L491 located in the undecapeptide or L1. Taken together, these results indicate that the interaction of L2 and L3 with the lipid bilayer play an important role in the affinity of PFO to cholesterol in membranes. The higher the tendency for an amino acid side chain to partition into the hydrophobic core of the membrane, the less cholesterol is required to trigger binding. In contrast, the undecapeptide and L1 seems to be involved in the specificity for cholesterol binding^32^, since substitutions of residues in these loops to Ala increased the Δmol% of the proteins. These results support the model where each of the loops in D4 play a distinct role during PFO binding, oligomerization, and insertion^32,37,52^.

**Table 2:** Comparison of the effect on cholesterol-dependent binding and free energy of the water-octanol partition for amino acid substitutions in D4.

ocation	%Conservation^a^	Modification	Δmol%	ΔΔG^b^
**undecapeptide**				
C459	86	A	4	0.5
**Loop 1**				
T490	100	V	4	−0.7
		A	5	0.3
L491	100	S	−2	1.7
		A	5	1.8
**Loop 2**				
S399	46	I	−1	−1.6
		A	1	0.0
A401	86	V	−1	−1.0
		G	1	0.7
V403	100	A	2	1.0
**Loop 3**				
D434	61	V	−4	−4.1
		A	−6	−3.1
		S	−6	−3.2
A437	79	V	−7	−1.0

^a^Based on sequence alignment of 28 CDCs^22^; ^b^Values obtained from^51^.

PFO binding depends on cholesterol exposure, which at a constant phospholipid composition increases with the cholesterol content^12^. It is expected that the incorporation (or removal) of cholesterol to (or from) the plasma membrane would modify cholesterol accessibility^15^, and consequently the binding of PFO to cells (as observed with liposomes). Based on the relative binding for the three PFO probes, the average cholesterol accessibility at the plasma membrane of RAW 264.7 cells seemed to remain fairly constant after treatments to add or remove cholesterol (Fig. 6). A similar binding pattern was observed with liposomes containing between 36 mol% and 39 mol% cholesterol (Fig. 3D). If cholesterol accessibility is lower than the one present in liposomes with 36 mol%, no binding of pPFO^D434S-Alexa488^ is expected. If it is higher than the one present in liposomes containing 39 mol%, binding of pPFO^T490A-Alexa488^ will be observed. Independently of the cholesterol content, no binding was observed for pPFO^T490A-Alexa488^ while both pPFO^D434S-Alexa488^ and pPFO^Alexa488^ were able to bind. This data suggest that despite variations in the total cholesterol content, RAW 264.7 cells maintained a rather constant average cholesterol accessibility at the plasma membrane, similar to the one observed in model membranes containing 1:1:1 SM:POPC:POPE and cholesterol in the 36–39 mol % range (Fig. 3D). How cholesterol accessibility is maintained constant in live cells when the overall cholesterol content varies remains unclear. To a certain level, accessibility could be maintained by adjusting the phospholipid composition (i.e., modifying the head-groups and/or number of double bonds in the acyl chains)^12,13^. Further studies would be required to establish the precise mechanism by which eukaryotic cells respond to changes in cholesterol content and regulate cholesterol accessibility at the plasma membrane.

In summary, we have obtained and characterized various non-lytic PFO derivatives that differentially bind to cholesterol-containing membranes and revealed novel features for the mechanism of PFO pore-formation. The energetics for the unfurling of the transmembrane β–hairpins of the toxin is modulated by two aromatic residues located in D3. The affinity of PFO for cholesterol-containing membranes was modulated by an amino acid substitution in D3 and single amino acid modifications in D4. We showed that the use of Δmol% cholesterol constitute an effective way to compare the affinity of different PFO derivatives for cholesterol. These cholesterol biosensors were used to show that cholesterol accessibility at the plasma membrane of live RAW264.7 cells was unchanged when the total cholesterol levels were modified using mβCD/cholesterol mixtures. Taken together, these results not only provide mechanistic insights for the cholesterol-dependent binding of PFO to membranes but also shed light on the selectivity and tunability of PFO-based probes to detect cholesterol accessibility.

## Methods

### Cell culture

RAW264.7 macrophage-like cells were cultured in Roswell Park Memorial Institute (RPMI) 1640: 10% FCS medium with 50 units/ml penicillin and 50 µg/ml streptomycin at 37 °C with 5% CO_2_. Cells were passaged at 70–80% confluence by removal of non-adherent cells and adherent removed by gently pipetting cell were re-plated with fresh media in a one to ten dilution.

### Hemolysis of sheep RBC

pPFO derivatives were dialyzed twice against 4 l of PBS (10 mM sodium phosphate, 1.74 mM potassium phosphate, 137 mM NaCl, 2.7 mM KCl, pH 7.4) for 4 h to exchange buffers and remove cryoprotectant glycerol. Washed sheep RBC were suspended in PBS to 1%. pPFO was serially diluted in a 96 well plate and then combined with an equal volume of the RBC suspension to a final concentration of 250 µl per well. This mixture was then incubated for 30 min at 37 °C. Non-lysed RBC were pelleted from the samples by centrifugation at 3500 × g for 10 min and 200 µl of supernatant was transferred to a new plate. The extent of hemoglobin release was quantified by measuring the absorbance at 540 nm of cell free supernatants. Controls were determined by osmotic shock of an identical amount of RBC with deionized water (100% lysis) or by incubation of RBC in the absence of PFO (0% lysis).

### Determination of Cell Viability

RAW264.7 cells were cultured as indicated above and non-adherent cells were eliminated. Adherent cells were then recovered by pipetting, counted using a hemocytometer, and washed two times with 1 ml of PBS containing 1% FCS. Aliquots of 1 million cells were washed in PBS 1% FCS and then incubated at 4 °C or 37 °C for 20 min with varied concentrations of the indicated PFO derivative in 100 μl of PBS 1% FCS. Cells were then washed and assessed for plasma membrane integrity by the use of the exclusion dye Trypan blue, and compared to a sample containing no protein.

### Flow cytometry

RAW264.7 cells were cultured, non-adherent cells were eliminated, and adherent cells scraped, counted, and re-suspended in RPMI 1640 medium. Aliquots of 1 million cells were then plated in a 24 well plate with RPMI medium containing serum and antibiotics in a final volume of 2 ml and grown overnight at 37 °C with 5% CO_2_. Cells were washed in serum free media and incubated for 2 h at 37 °C with 5% CO_2_. Cells were washed with PBS and then incubated at 37 °C for 30 min in 0.5 ml of PBS containing 5 mM mβCD (Sigma) complexed with the indicated concentration of cholesterol. The cells were then washed twice with PBS and incubated at 4 °C for 20 min with 0.5 μM of the indicated PFO derivative (10% labeled with Alexa488) in 100 μl of PBS.

### Preparation of lipids and liposomes

Large unilamellar vesicles were generated as described previously^25,50^. Briefly, equimolar mixtures of 1-palmitoyl-2-oleoyl-sn-glycero-3-phosphocholine (POPC), 1-palmitoyl-2-oleoyl-sn-glycero-3-phosphoethanolamine (POPE), and sphingomyelin (SM, brain, porcine), were combined with the indicated amount of cholesterol (5-cholesten-3β-ol). The re-suspended lipid film was extruded through a 0.1 μm filter 21 times using a buffer solution containing Hepes 50 mM pH 7.5 and NaCl 100 mM.

### Assay for PFO binding to liposomes

Binding assay was done using the change in the Trp emission produced by the binding of PFO to cholesterol containing membranes as described previously^25^. The fraction of protein bound was determined as (F − F_0_)/(F_f_ − F_0_), where F_f_ is the emission intensity when all the protein is bound. Total lipid concentration was 0.2 mM.

### Preparation of PFO derivatives and labeling

The expression, purification, and labeling with malemide derivative of Alexa 488 of the PFO derivatives were done as described previously^25^. Mutagenesis of PFO was done using the QuickChange (Stratagene) procedure.

### Sequential binding of PFO derivatives determined using intrinsic tryptophan fluorescence

The consecutive binding of two different PFO derivatives with different cholesterol thresholds was tested on liposomes containing 36 mol % cholesterol. The first PFO derivative was added to a cuvette and Trp emission determined (F_0_). Liposomes were then added and the sample was incubated for 20 min at 37 °C. Bound PFO was determined by the net increase in Trp emission (after blank subtraction and dilution corrections) that follows the interaction with the membrane (F)^13^. A second PFO derivative was then added to the same cuvette and incubated for another 20 min at 37 °C and the final fluorescence was determined. The Trp emission of the sample was recorded, and the fluorescence increase (F) calculated as the difference between the emission before and after incubation with the second PFO derivative. The Trp emission corresponding to the unbound second derivative (F_0_) was determined in a separate cuvette in the absence of membranes. The binding of the second PFO derivative was then determined using the increase in the Trp emission as described above.

### Preparation of mβCD complexed with cholesterol

A solution of cholesterol in methanol-chloroform (2:1 v/v) was added dropwise to a stirred solution of mβCD in PBS on a water bath (80 °C)^53^. Once the sterol was added to the mβCD solution, a cloudy precipitate formed. Complete dissolution of the sterol was achieved after allowing the mixture to stir for about 30–45 min. The samples were cooled down to 4 °C and centrifuged for 30 min at 25000 × g at this temperature. The supernatant of each sample was stored at 4 °C for up to 2 weeks.

### Steady-State Fluorescence Spectroscopy

Steady-state fluorescence measurements were taken using a Fluorolog-3 photon-counting spectrofluorometer as described previously^13^. Samples were equilibrated at 25 °C before measurements.

## Electronic supplementary material

Supplementary Information
