# Supplementary material for: Mechanistic Insights into the Cholesterol-dependent Binding of Perfringolysin O-based Probes and Cell Membranes
Source: Sci Rep. 2017 Oct 23;7:13793. doi: 10.1038/s41598-017-14002-x (PMC5653841; doi:10.1038/s41598-017-14002-x)
Supplement: Supplementary file 1 — Supplementary Information [file 41598_2017_14002_MOESM1_ESM.doc]

**Mechanistic Insights into the Cholesterol-dependent Binding**

**of Perfringolysin O-based Probes and Cell Membranes**

Benjamin B. Johnson, Mariana Breña, Juan Anguita, and Alejandro P. Heuck

**Supplementary Information**

**FIGURE 1S. Labeling of pPFO with Alexa488 does not affect the cholesterol threshold.** Cholesterol-dependent binding isotherm for pPFO. pPFO (open circles) and pPFO^Alexa488^ (filled circles) were added to a final concentration of 0.2 μM, and incubated with liposomes of varying cholesterol concentrations and a constant 1:1:1 molar ratio of POPC, POPE, and SM (final total lipid concentration of 0.2 mM). PFO binding was determined using the intrinsic Trp emission before (F_0_) and after addition of liposomes (F) at 20 ^o^C. Values at each cholesterol mol % are the average of two measurements and the errors bars indicate the range.

**FIGURE 2S. Cholesterol-dependent binding isotherms for pPFO**. pPFO at the indicated final concentration was incubated in 50 mM buffer Hepes pH 7.5, 100 mM NaCl, 1mM DTT, and 0.5 mM EDTA with liposomes of varying cholesterol concentrations and a constant 1:1:1 molar ratio of POPC, POPE, and SM (final total lipid concentration of 50 μM). Samples were incubated for 20 min at 37 °C in a final volume of 300 μL. PFO binding was determined using the intrinsic Trp emission at 20 ^o^C before (F_0_) and after addition of liposomes (F) as described in methods. Values at each cholesterol mol % are the average of two measurements and the errors bars indicate the range.
